# Climate of the Western Hemisphere

**Published:** 1915-10

**Authors:** 


**Climate of the Western Hemisphere.** Rainfall, inches. Prudential Life Ins. Co.

| Country | City | Jan. to March | April to June | July to Sept. | Oct. to Dec. | Annuel |
|---|---|---|---|---|---|---|
| Panama | Colon | 7.7 | 27.8 | 27.8 | 55.6 | 128.9 |
| Saint Vincent | Kingston | 16.1 | 25.8 | 35.8 | 30.3 | 108.0 |
| Brazil | Para | 41.0 | 31.8 | 15.6 | 13.9 | 102.3 |
| Dutch Guiana | Paramaribo | 23.5 | 33.6 | 19.0 | 16.3 | 92.4 |
| British Guiana | Georgetown | 22.5 | 30.5 | 19.5 | 19.4 | 91.9 |
| Brazil | Manaos | 31.2 | 25.6 | 6.3 | 20.6 | 83.7 |
| British Honduras | Belize | 12.1 | 23.1 | 22.7 | 24.7 | 82.6 |
| Grenada | Dichmond Hill | 10.2 | 16.7 | 27.6 | 23.1 | 77.6 |
| Philippine Islands | Manila | 2.3 | 15.1 | 43.7 | 15.1 | 76.2 |
| Costa Rica | San Jose | 1.6 | 19.7 | 30.1 | 17.7 | 69.1 |
| Brazil | Bahia | 11.4 | 29.5 | 13.5 | 11.7 | 66.1 |
| Trinidad | Port of Spain | 6.4 | 14.1 | 26.2 | 18.4 | 65.1 |
| Porto Rico | San Juan | 9.8 | 14.8 | 20.3 | 19.1 | 64.0 |
| Colombia | Bogota | 11.7 | 19.3 | 8.8 | 23.6 | 63.4 |
| U. S. | New Orleans | 11.0 | 19.2 | 20.5 | 10.3 | 61.0 |
| Barbados | Bridgetown | 7.3 | 11.0 | 19.2 | 20.3 | 57.8 |
| Brazil | Curityba | 17.3 | 11.5 | 11.1 | 17.6 | 57.5 |
| Newfoundland | St. Johns | 16.1 | 12.9 | 10.0 | 18.4 | 57.4 |
| Canada | Halifax | 15.6 | 12.9 | 12.2 | 16.5 | 57.2 |
| Canada | Vancouver | 19.0 | 7.6 | 6.4 | 24.0 | 57.0 |
| Brazil | Bello Horizonte | 25.7 | 2.4 | 4.5 | 23.6 | 56.2 |
| Bermuda | Hamilton | 12.7 | 11.3 | 13.5 | 14.4 | 51.9 |
| Cuba | Babana | 6.8 | 14.5 | 17.8 | 12.6 | 51.7 |
| Brazil | Rie de Janeiro | 20.5 | 8.6 | 6.3 | 14.8 | 50.2 |
| Paraguay | Asuncion | 16.9 | 8.7 | 6.5 | 15.2 | 47.3 |
| Canada | St. John | 11.6 | 10.7 | 10.2 | 12.0 | 44.5 |
| Ecuador | Quito | 13.5 | 13.9 | 5.3 | 11.4 | 44.1 |
| Canada | Montreal | 11.4 | 9.6 | 10.2 | 9.7 | 40.9 |
| U. S. | Washington | 8.5 | 11.1 | 10.9 | 8.7 | 39.2 |
| Peru | Cuzco | 20.4 | 4.5 | 2.2 | 11.6 | 38.7 |
| Argentina | Tucuman | 19.1 | 6.3 | 1.3 | 11.7 | 38.4 |
| Argentina | Buenos Aires | 10.2 | 8.6 | 7.6 | 10.4 | 36.8 |
| Jamaica | Kingston | 2.7 | 10.0 | 9.9 | 12.9 | 35.5 |
| U. S. | Chicago | 6.8 | 10.8 | 10.5 | 6.7 | 34.8 |
| U. S. | Boston | 8.7 | 8.1 | 8.7 | 9.0 | 34.5 |
| Venezuela | Caracas | 1.8 | 8.0 | 12.8 | 9.4 | 32.0 |
| Hawaii | Honolulu | 11.0 | 5.9 | 4.2 | 10.0 | 31.1 |
| Canada | Toronto | 6.1 | 8.7 | 8.9 | 7.1 | 30.8 |
| Uruguay | Montevideo | 7.5 | 7.6 | 8.0 | 7.5 | 30.6 |
| Falkland Islands | Port Stanley | 7.0 | 7.3 | 5.7 | 6.5 | 36.5 |
| Greenland | Godthaab | 5.9 | 4.8 | 8.8 | 6.5 | 26.0 |
| Nicaragua | San Juan del Norte | 4.1 | 5.5 | 7.9 | 8.4 | 25.9 |
| Chile | Santiago | 0.3 | 10.2 | 12.6 | 1.4 | 24.5 |
| Mexico | Mexico City | 0.9 | 6.5 | 14.0 | 2.5 | 23.9 |
| U. S. | San Francisco | 15.8 | 0.8 | 0.2 | 4.9 | 21.7 |
| Argentina | Bahia Blanca | 6.6 | 4.4 | 3.6 | 6.3 | 20.9 |
| Canada | Edmonton | 1.4 | 6.3 | 6.0 | 2.8 | 16.5 |
| U. S. | Los Angeles | 11.0 | 0.3 | 0.5 | 3.3 | 15.1 |
| Canada | Dawson | 2.2 | 2.9 | 5.5 | 4.1 | 14.7 |
| U. S. | Denver | 1.8 | 4.9 | 5.6 | 1.9 | 14.2 |
| Alaska | Nome | 2.1 | 1.8 | 7.5 | 2.6 | 14.0 |
| Chile | Punta Arenas | 1.9 | 5.5 | 2.9 | 1.2 | 11.5 |
| Bolivia | La Paz | 1.1 | 0.2 | 0.1 | 0.7 | 2.1 |
| Peru | Lima | 0.1 | 0.2 | 1.3 | 0.2 | 1.8 |
| Chile | Arica | 0.0 | 0.0 | 0.0 | 0.0 | 0.0 |

